# A dynamic vascular–inflammatory–fibrotic loop in systemic sclerosis: an integrative immuno-vascular framework

**DOI:** 10.3389/fimmu.2026.1784630

**Published:** 2026-05-01

**Authors:** Razvan Constantin Ionitescu

**Affiliations:** Department of Rheumatology, MedArt Cliniq, Râmnicu-Vâlcea, Romania

**Keywords:** disease heterogeneity, dynamic disease modeling, immune network geometry, nailfold videocapillaroscopy, phase-adapted therapy, systemic sclerosis, vascular-immune-fibrotic loop

## Abstract

**Background:**

Systemic sclerosis (SSc) is characterized by a complex and heterogeneous interplay between vascular dysfunction, immune activation, and progressive fibrosis. Despite advances in molecular characterization, therapeutic responses remain inconsistent and often transient, suggesting that disease persistence arises from dynamic interactions rather than isolated pathogenic pathways.

**Objective:**

To propose a clinically grounded conceptual framework that integrates vascular, immune, and fibrotic processes into a dynamic loop model of SSc, derived from longitudinal clinical observations and contextualized within existing immunological and vascular literature.

**Methods:**

This integrative framework is informed by long-term clinical follow-up, nailfold videocapillaroscopy patterns, immune biomarker profiling, and outcome analyses reported in SSc cohorts. Observations are organized into operational domains and translated into testable predictions reflecting shifts in pathway dominance over time.

**Results:**

The proposed model conceptualizes SSc as a self-sustaining dynamic loop in which vascular injury, immune signaling, and fibrotic remodeling interact non-linearly. Capillaroscopic patterns emerge as relatively stable structural readouts of disease phase, while immune and oxidative biomarkers reflect more dynamic functional states. This framework accounts for clinical heterogeneity, variable therapeutic responses, and the limited durability of single-target interventions.

**Conclusions:**

Rather than representing a static disease entity, SSc can be understood as a dynamic system governed by shifting pathway dominance within a persistent loop architecture. This model provides a unifying structure for interpreting disease heterogeneity and supports the development of stage-adapted, combination therapeutic strategies.

## Highlights

- Systemic sclerosis is conceptualized as a self-reinforcing vascular–immune–fibrotic loop rather than a linear cascade.
- Vascular dysfunction, immune activation and fibrosis operate as interdependent domains with dynamic feedback.
- Immune activity is organized through cytokine network geometry rather than isolated mediator dominance.
- Fibrosis acts as an active signaling compartment sustaining disease persistence.
- Clinical heterogeneity reflects differential axis weighting within a shared pathogenic architecture.

## 1 Introduction

SSc represents one of the most complex immune-mediated connective tissue diseases, characterized by the coexistence and mutual reinforcement of microvascular dysfunction, immune dysregulation and progressive fibrosis affecting the skin and internal organs (1–3). Despite decades of intensive research, the pathogenic architecture of SSc remains only partially understood, largely due to the difficulty of integrating these domains into a unified explanatory framework.

Historically, three major pathogenetic paradigms have dominated the field:

- First, vascular-centric models emphasized endothelial injury, vasospasm and microangiopathy as primary events driving tissue ischemia and organ damage (4–6).
- Second, immune-centric models focused on autoimmunity, cytokine imbalance and adaptive immune polarization, particularly involving T helper cell subsets and B-cell–mediated autoantibody production (7–9).
- Third, fibroblast-centric models described dysregulated extracellular matrix deposition and fibroblast activation as the central drivers of irreversible tissue sclerosis (10–12).

While each of these paradigms has generated valuable insights, none fully explains several defining features of SSc, including:

- the persistence of disease activity over decades,
- the striking heterogeneity of clinical phenotypes,
- the frequent dissociation between inflammatory markers and fibrotic progression,
- the limited durability of single-target therapeutic interventions (10, 11, 13).

Over the last two decades, accumulating experimental, clinical and imaging data have increasingly suggested that SSc cannot be adequately described as a linear cascade progressing from inflammation to fibrosis, but rather as a dynamic system in which vascular injury, immune activation and fibrotic remodeling continuously interact and reinforce one another (1–3).

Nailfold videocapillaroscopy has demonstrated that microvascular abnormalities often precede overt fibrosis and persist throughout disease evolution. Immunologic studies reveal sustained cytokine activity even in clinically fibrotic stages, while fibrotic tissue remodeling further compromises vascular architecture, perpetuating hypoxia, oxidative stress and immune activation. These observations collectively argue against rigid phase-based models and instead support a framework of self-sustaining pathogenic feedback loops (4, 11, 14, 15).

Despite this recognition, most existing conceptual models remain fragmented, often depicting disease evolution as sequential stages that inadequately reflect real-world clinical behavior. Few models explicitly integrate vascular biology, immune network organization and fibrotic mechanics into a single coherent construct.

This work should be interpreted as a conceptual and integrative framework derived from long-term longitudinal clinical observations and targeted literature integration, rather than as a hypothesis-driven experimental study or a systematic review of recent advances.

Rather than reiterating linear causality, this framework focuses on how shifting dominance between vascular, immune and fibrotic axes shapes disease behavior over time.

## 2 Rationale and conceptual aim

In this context, we propose an integrative conceptual framework that captures the dynamic interplay between vascular dysfunction, immune activation and fibrosis in SSc, developed through long-term clinical observation, capillaroscopic assessment, immunologic reasoning and synthesis of foundational and contemporary experimental and translational literature.

Rather than positioning vascular damage, immune dysregulation or fibrosis as isolated or hierarchical events, this model conceptualizes SSc as a self-reinforcing pathogenic loop in which each domain acts both as a driver and a consequence of the others. Importantly, pathogenic dominance may shift over time without collapsing the system, accounting for disease persistence, clinical heterogeneity and the limited durability of single-target therapeutic approaches.

The framework is articulated through two complementary representations:

(1) a dynamic loop model describing bidirectional interactions between endothelial dysfunction, immune activation and fibroblast-driven tissue remodeling, and (2) an immunological geometry model capturing cytokine-driven axis interactions and network stability.

Together, these representations aim to move beyond reductionist views and provide a systems-level interpretation of SSc pathogenesis.

**Figure 1:**
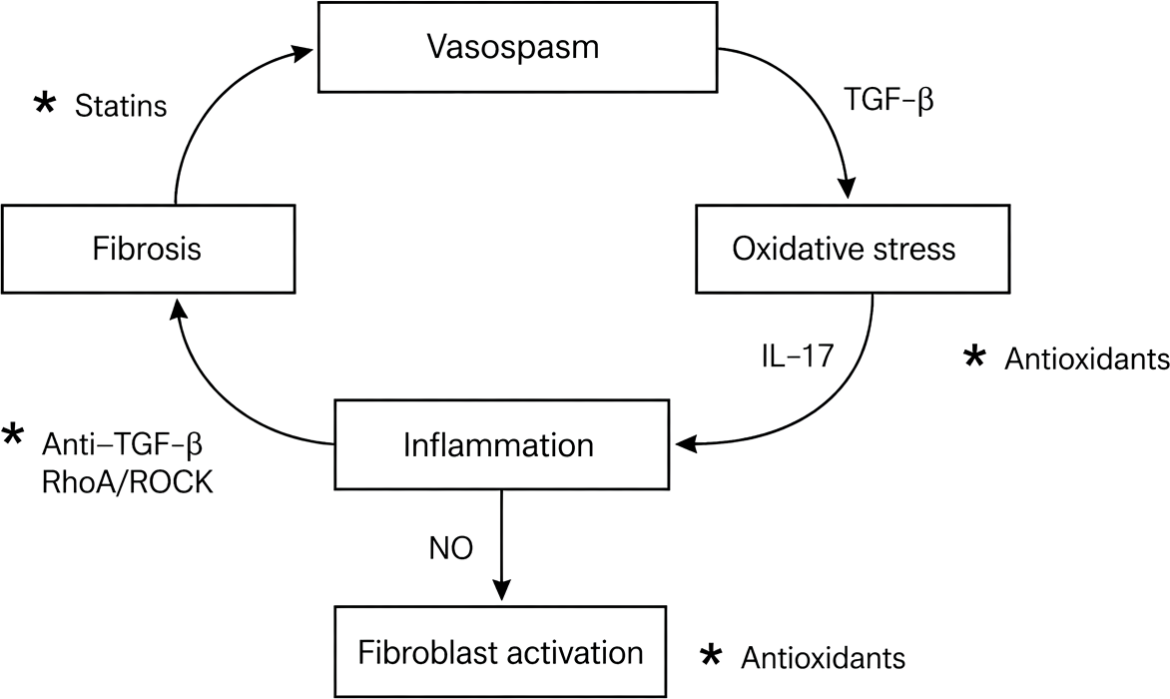
Dynamic vascular–inflammatory–fibrotic loop in systemic sclerosis (SSc). Conceptual representation of the vascular–inflammatory–fibrotic feedback loop in SSc. Arrows indicate dominant directional influences between vasospasm, oxidative stress, inflammation, and fibrosis. Asterisks (*) denote potential therapeutic intervention points that modulate loop dynamics rather than representing intrinsic biological components.

To integrate these observations into a coherent pathogenic structure, we propose a dynamic vascular–inflammatory–fibrotic loop that conceptualizes SSc as a self-sustaining system rather than a linear cascade (**Figure 1**).

Conceptual explanation:

**Figure 1** introduces the core conceptual architecture of the proposed model. It visually anchors the manuscript by presenting SSc as a dynamic, self-perpetuating loop rather than a linear disease sequence. The figure integrates recurrent vascular stress, immune amplification and fibrotic stabilization into a unified pathogenic circuit, serving as the structural reference point for subsequent mechanistic and clinical interpretation.

At the vascular level, recurrent vasospasm emerges as an early and functionally central driver of endothelial dysfunction and tissue hypoxia. These perturbations promote oxidative stress through increased generation of reactive oxygen species, thereby amplifying inflammatory signaling and immune dysregulation. Transforming growth factor-β (TGF-β) acts as a critical molecular link between oxidative stress and fibrotic pathways, driving fibroblast activation and extracellular matrix accumulation.

Inflammation occupies a central integrative role within the loop, acting as a convergence point for signals derived from oxidative stress, immune activation (including IL-17 (IL-17A)–associated pathways) and endothelial dysfunction. Through this integrative function, inflammatory signaling amplifies cross-domain interactions rather than operating as an isolated process. Inflammatory mediators further impair nitric oxide (NO) bioavailability, thereby exacerbating vasospasm and perpetuating vascular injury.

Downstream of inflammation, sustained fibroblast activation drives progressive fibrosis, which in turn feeds back on vascular structure and function, reinforcing the pathogenic cycle and stabilizing the system in a self-perpetuating state. This configuration emphasizes persistence through interaction rather than progression through sequence.

### 2.1 Operationalization and testable components

To enable empirical application, the proposed framework is translated into measurable biological domains that reflect the dynamic structure of the pathogenic loop.

These domains include microvascular structure and function, immune activation, oxidative stress and fibrotic remodeling (7, 8, 10, 11, 16, 17). Each domain can be approximated through clinically accessible parameters, enabling integration of mechanistic insight with real-world patient evaluation (**Table 1**).

**Table 1:** Operational domains, modulable nodes and testable readouts derived from the loop-based model:.

Domain	Primary functional node	Representative processes	Measurable readouts (examples)	Predicted behavior within the loop	Interpretive value
*Vascular*	Vasospasm/endothelial dysfunction	Altered vascular tone, endothelial injury, impaired perfusion.	Raynaud frequency/severity, nailfold videocapillaroscopy parameters, clinical vascular scores.	Early and persistent instability; partial modulation may transiently attenuate downstream domains.	Identifies vascular entry and persistence nodes.
*Oxidative stress*	Redox imbalance	ROS generation, endothelial–immune amplification.	Oxidative stress biomarkers (when available), indirect inflammatory surrogates.	Amplifies inflammatory signaling rather than acting as an isolated compartment.	Functions as a loop amplifier rather than a terminal endpoint.
*Immune/inflammatory*	Inflammatory amplification	Cytokine-mediated immune–stromal crosstalk (including IL-17–associated pathways).	CRP, ESR, selected cytokines (IL-6, IL-17A).	Dynamic and state-dependent; reinforces vascular injury and fibrotic signaling.	Reflects immune plasticity and non-linearity.
*Fibrotic*	Fibroblast activation/matrix signaling	TGF-β–linked profibrotic pathways, extracellular matrix deposition.	Clinical fibrotic burden, TGF-β–related markers, skin/lung involvement.	May persist despite inflammatory attenuation; exhibits feed-forward behavior.	Explains dissociation between inflammation and fibrosis.
*Interventional (hypothesized)*	Modulable loop nodes*	Vascular, oxidative, or profibrotic perturbation.	Therapy-associated clinical/bio-marker shifts.	Partial or transient responses unless multiple domains are targeted.	Supports multi-node or stage-adaptive strategies.
*System-level configuration*	Loop dominance state	Relative weighting of vascular, immune and fibrotic domains.	Integrated vascular-immune-fibrotic profiles.	Distinct equilibrium configuration underlying clinical heterogeneity.	Moves beyond linear staging toward dynamic phenotypes.

*Modulable nodes are indicated in **Figure 1** by an asterisk and represent hypothesized points of loop perturbation rather than established therapeutic effects (11, 22).

Microvascular dynamics are assessed through nailfold videocapillaroscopy, capturing structural alterations and perfusion abnormalities that reflect early vascular instability (4, 5). Immune activation is represented by circulating inflammatory markers, including CRP, ESR and selected cytokines, which provide a composite signal of inflammatory amplification(7, 8, 16). Oxidative stress, although less directly measurable, can be inferred through surrogate biomarkers and its coupling with inflammatory activity (18). Fibrotic remodeling is captured through clinical indicators of organ involvement and TGF-β–associated pathways, reflecting downstream tissue reorganization and matrix deposition (10, 11, 17).

By mapping these measurable domains onto the conceptual loop, the framework enables the generation of testable predictions regarding disease trajectory, domain dominance and therapeutic responsiveness (19–21). Longitudinal assessment of these parameters allows dynamic tracking of shifts within the loop, rather than static classification of disease stage.

To enhance falsifiability and facilitate experimental validation, specific nodes within the loop are identified as potentially modulable components. These nodes, indicated in **Figure 1** by an asterisk (*), represent hypothesized points of loop perturbation rather than established therapeutic targets (22, 23).

Within this conceptual structure, different classes of interventions can be interpreted according to their position within the loop (22–24). Statins are hypothesized to modulate the vasospasm–endothelial dysfunction axis through pleiotropic vascular effects, including improved nitric oxide bioavailability and reduction of oxidative stress. Antioxidants may attenuate oxidative stress and its downstream amplification of inflammatory signaling, thereby indirectly influencing both immune activation and fibroblast behavior. Inhibition of TGF-β signaling and RhoA/ROCK pathways is proposed to interfere with the inflammation–fibrosis interface, potentially reducing fibroblast activation and extracellular matrix deposition.

Importantly, the model does not assume that modulation of a single node is sufficient to disrupt disease progression. Instead, it predicts that isolated interventions may yield only transient effects, whereas durable responses are more likely to require combination or phase-adapted strategies targeting multiple interconnected domains (22–24).

### 2.2 Clinical implications and translational relevance

Building on the operational domains and testable components described above, the proposed framework can be extended toward clinical interpretation.

Rather than classifying patients into fixed stages, the model suggests that individuals can be characterized by the relative dominance of vascular, inflammatory or fibrotic components within a dynamic pathogenic loop. This perspective may help explain why patients with similar clinical phenotypes often exhibit divergent disease trajectories and variable therapeutic responses.

The framework also enables a phase-oriented interpretation of disease expression. Periods of vascular predominance may be characterized by microvascular instability and endothelial dysfunction, whereas immune-dominant phases reflect heightened cytokine activity and inflammatory amplification. In later stages, fibrotic processes may become relatively autonomous, maintaining disease progression despite attenuation of upstream inflammatory signals.

From a therapeutic perspective, the model suggests that interventions targeting a single pathogenic axis may lead to partial or transient responses when other domains remain active. Therapeutic responses can therefore be interpreted according to their alignment with the dominant pathogenic domain. Strategies addressing multiple interconnected components of the loop may result in more sustained clinical benefit.

Importantly, this framework is not intended to prescribe treatment algorithms, but rather to support clinical reasoning in complex or heterogeneous cases, including those with discordant features or apparent therapeutic resistance.

From a clinical monitoring perspective, the model supports longitudinal assessment of disease dynamics through integrated evaluation of vascular, immune and fibrotic domains, allowing a more adaptive interpretation of disease trajectory over time.

Overall, the loop-based framework provides a structured interpretative tool linking pathogenic domain dominance to clinical behavior, offering a conceptual bridge between mechanistic understanding and real-world decision-making in SSc.

### 2.3 Scope and boundaries of the model

This framework is intended as a conceptual synthesis integrating established vascular, immunological and fibrotic mechanisms in SSc. It does not aim to provide an exhaustive molecular description of disease pathogenesis, nor to replace detailed mechanistic models of individual pathways.

Rather, the model focuses on system-level interactions between key pathogenic domains, emphasizing their dynamic coupling over time. While not disease-specific at the molecular level, the framework is particularly relevant to SSc due to the early and prominent involvement of the microvasculature and its interaction with immune activation and fibrotic remodeling.

Within this loop-based structure, model-derived predictions emerge from interactions between domains rather than isolated pathways. The framework therefore generates hypotheses that are inherently relational and longitudinal, reflecting coordinated shifts across vascular, immune and fibrotic compartments.

Specifically, the model predicts that measurable changes within one domain will be associated with corresponding adaptations in others, consistent with system-level coupling rather than independent compartmental activity. These interactions may exhibit temporal dissociation, reflecting disease stage, compensatory mechanisms or therapeutic modulation.

Importantly, the framework does not assume linear causality or uniform directionality. Instead, it emphasizes feedback reinforcement, threshold effects and non-linear transitions between loop configurations. As such, its predictions are designed to be falsifiable through longitudinal clinical observation, integrated biomarker assessment and dynamic vascular evaluation.

The following section outlines model-derived predictions as operational extensions of this conceptual framework, intended to support empirical testing and to evaluate whether targeted perturbation of loop components can meaningfully influence disease trajectories.

### 2.4 Model-derived predictions

Importantly, model-derived predictions do not assume linear causality or synchronous evolution of biological domains, but rather emphasize non-linear, state-dependent trajectories that require longitudinal assessment for accurate interpretation (25).

Prediction 1 — Vascular node modulation should affect downstream loop readouts.

If vasospasm/endothelial dysfunction represents a functional entry and persistence node of the loop, then interventions associated with improved endothelial function should be accompanied by measurable attenuation of downstream loop activity.

- Measure: vascular tone proxies (Raynaud frequency/severity), nailfold videocapillaroscopy parameters, oxidative stress surrogates (when available) and inflammatory markers (CRP/ESR ± selected cytokines).
- Would weaken the model: consistent absence of downstream change despite demonstrable improvement in vascular function.

Prediction 2 — Oxidative stress behaves as an amplification node rather than a passive byproduct.

If oxidative stress functions as an amplification node within the loop, then its fluctuations should align more closely with inflammatory activation than with fibrotic burden at single time points.

- Measure: oxidative stress biomarkers (when available) alongside CRP/ESR and selected cytokines.
- Would weaken the model: oxidative stress consistently tracking fibrosis alone, independent of inflammatory activity.

Prediction 3 — IL-17–associated signaling acts as an amplifier within inflammatory coupling.

IL-17–associated pathways are predicted to function primarily as amplification components within the inflammatory domain, reinforcing endothelial–immune–stromal crosstalk rather than acting as single dominant drivers (12).

- Measure: IL-17A (± IL-6) in relation to inflammatory activity and vascular instability measures.
- Would weaken the model: absence of any relationship between IL-17–axis activity and inflammatory or vascular loop behavior across patients or over time.

Prediction 4 — TGF-β–linked fibroblast activation may persist despite inflammatory attenuation.

Fibrosis represents an active signaling compartment within the loop, then reduction of systemic inflammatory markers will not uniformly translate into reduction of profibrotic activity when TGF-β–linked signaling remains dominant.

- Measure: CRP/ESR (± inflammatory cytokines) versus profibrotic indicators (TGF-β–related markers and/or fibrosis biomarkers; clinical fibrotic burden).
- Would weaken the model: robust, uniform parallel decline of fibrotic readouts whenever inflammation decreases.

Prediction 5 — Node-specific perturbations yield partial or transient responses unless loop dominance is shifted.

Targeting a single node (vascular, oxidative, or profibrotic) is predicted to produce partial or transient improvement if the overall loop configuration remains stable due to compensatory activity in other domains.

- Measure: short-term clinical or biomarker improvement followed by plateauing or redistribution of activity across domains.
- Would weaken the model: consistent, durable collapse of multi-domain activity following isolated single-node modulation.

Prediction 6 — Non-linearity: domain readouts may evolve asynchronously.

Because the loop is dynamic and state-dependent, vascular, immune and fibrotic readouts are predicted to evolve asynchronously, particularly under therapeutic intervention or during disease-state transitions.

- Measure: longitudinal trajectories showing improvement in one domain without immediate concordant change in others.
- Would weaken the model: strict, consistent synchronous evolution of all domains across patients.

Prediction 7 — Clinical heterogeneity reflects loop dominance states rather than a single severity continuum.

Distinct SSc phenotypes (vascular-dominant, immune-dominant, fibrotic-dominant) are predicted to reflect different equilibrium configurations (dominance states) within the same pathogenic loop, rather than purely time-dependent severity stages.

- Measure: clustering of patients by combined vascular, immune and fibrotic profiles rather than disease duration alone.
- Would weaken the model: inability to identify reproducible dominance patterns beyond a single severity axis.

Prediction 8 — Axis re-weighting rather than single-cytokine dominance.

If immune activation in SSc is organized along interacting cytokine axes rather than isolated pathways then disease evolution and therapeutic perturbation should be reflected by dynamic re-weighting of axis dominance rather than uniform suppression of individual cytokines (11, 12, 19, 22, 26).This is consistent with systems-level observations indicating that therapeutic effects in immune-mediated diseases often reflect reconfiguration of network dominance rather than sustained suppression of single mediators (6).

Measure:

- longitudinal changes in relative coupling between pro-inflammatory (e.g., IL-6/IL-17–associated), profibrotic (e.g., TGF-β/IL-4–associated) and counter-regulatory (e.g., IFN-γ/IL-10–associated) axes.

Would weaken the model:

- stable axis relationships across disease stages and treatments or dominance of single cytokines independent of network context.

**Figure 2:**
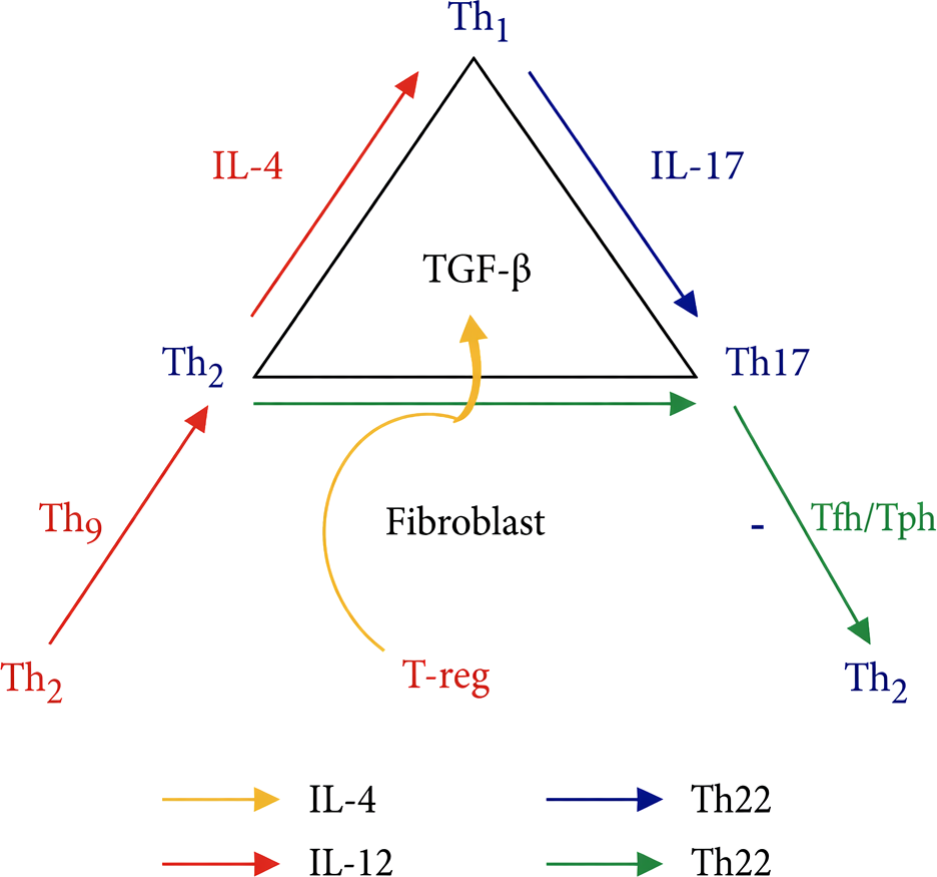
Updated immunological geometry of systemic sclerosis (SSc). This schematic presents a heuristic immunological geometry illustrating dominant cytokine axes and stabilizing interactions in SSc. The figure is not intended as a comprehensive mechanistic map, but as a relational framework highlighting amplification, counterbalance and domain dominance relevant to disease persistence and clinical heterogeneity. Immune activation in SSc is organized along interacting axes rather than isolated cytokine drivers, including pro-inflammatory (e.g., IL-6/IL-17–associated pathways), profibrotic (e.g., TGF-β and IL-4–associated signaling) and counter-regulatory pathways. The TGF-β–centered axis integrates fibroblast activation with context-dependent immunoregulatory signals. Geometric relationships reflect relative dominance and stabilization among immune domains rather than linear causality. Overall, the figure provides a conceptual organizing tool to interpret immune heterogeneity and to guide hypothesis generation and testable predictions rather than to assert definitive mechanistic pathways.

This axis-based re-weighting concept is schematically illustrated in **Figure 2** (11).

Together, these predictions operationalize the proposed loop-based model by defining testable, state-dependent relationships across vascular, immune, oxidative and fibrotic domains.

In the following section we integrate these elements into a unified conceptual framework that illustrates how dynamic interactions within the loop give rise to disease persistence, non-linearity and clinical heterogeneity in SSc.

## 3 Results/conceptual framework

### 3.1 Conceptual integration of the dynamic vascular–inflammatory–fibrotic loop *(SSc conceptualized as a self-sustaining pathogenic loop).*

The integrative analysis of vascular pathology, immune dysregulation and fibrotic remodeling supports the interpretation of SSc as a self-sustaining pathogenic loop rather than a linear or stage-restricted disease process.

Within this framework, vascular dysfunction, immune activation and fibrosis are not sequential phenomena but interdependent biological domains, each capable of initiating, amplifying and stabilizing the others over time (19–21). This dynamic interaction generates a closed pathogenic circuit that explains disease persistence, clinical heterogeneity and resistance to single-axis therapeutic strategies.

**Figure 1** schematically illustrates this dynamic vascular–inflammatory–fibrotic loop, integrating recurrent endothelial stress, immune amplification and fibroblast-driven tissue remodeling into a single conceptual architecture.

### 3.2 Vascular destabilization as a persistent driver of immune dysregulation

Endothelial dysfunction in SSc is characterized by early and persistent microvascular abnormalities, including capillary rarefaction and architectural disorganization, which can be longitudinally assessed by nailfold videocapillaroscopy (4, 15).

Microvascular rarefaction and capillary loss precede and predict irreversible fibrotic progression, positioning vascular injury as an early driver rather than a secondary consequence of SSc (11).

The first key result emerging from this integrative model is *the repositioning* of vascular injury from a transient initiating event to a persistent disease-driving axis.

Endothelial dysfunction in SSc is characterized by:

- impaired nitric oxide bioavailability,
- increased endothelin-1 signaling,
- oxidative stress and ischemia–reperfusion injury,
- structural capillary rarefaction.

Rather than resolving after immune activation, vascular damage continuously reshapes immune behavior by altering leukocyte trafficking, antigen presentation thresholds and cytokine gradients. Endothelial cells thus act as active immunomodulatory platforms, sustaining chronic immune activation even in advanced fibrotic stages.

This observation explains why vascular manifestations often precede, accompany and persist beyond inflammatory or fibrotic phases.

### 3.3 Immune network stabilization through cytokine geometry

A second central result is the identification of cytokine network geometry as a stabilizing force underlying immune dysregulation.

Sustained immune activation promotes endothelial dysfunction and maladaptive vascular remodeling, creating a permissive microenvironment for progressive fibroblast activation and tissue fibrosis (10).

To further explain the persistence and adaptability of immune dysregulation, we introduce an updated immunological geometry model that integrates cytokine axes and cellular subsets involved in SSc. Rather than reflecting linear cytokine causality, this framework emphasizes network configuration and domain dominance within the immune system (**Figure 2**).

**Figure 2** depicts this updated immunological geometry, in which cytokines are positioned relationally rather than hierarchically, emphasizing balance, asymmetry and redundancy rather than linear causality.

IL-17–associated signaling has been shown to play a central role in sustaining inflammatory amplification and immune–stromal crosstalk in SSc, contributing to disease persistence rather than acting as a transient inflammatory signal (9, 12, 27, 28).

This geometric organization explains:

- persistence of immune activation despite fluctuating cytokine levels,
- partial or transient responses to targeted biologics,
- coexistence of inflammatory and fibrotic signals across disease stages.

### 3.4 Fibrosis as an active biological axis

Contrary to classical models describing fibrosis as an irreversible endpoint, the present framework identifies fibrosis as an active biological axis that feeds back into vascular and immune compartments.

Emerging evidence indicates that activated fibroblasts in SSc actively modulate immune responses and vascular function through persistent profibrotic signaling, extracellular matrix–derived cues and mechanical feedback, thereby functioning as a dynamic disease-sustaining compartment rather than a terminal outcome (11, 12, 18).

Activated fibroblasts:

- generate mechanical stiffness,
- alter tissue oxygenation,
- produce profibrotic mediators that reinforce endothelial injury,
- amplify immune activation through stromal–immune crosstalk.

Extracellular matrix remodeling thus becomes a signal-generating process, rather than merely structural scarring (18, 29).

This insight explains why fibrotic progression may continue in the absence of overt inflammation and why anti-inflammatory therapies alone often fail to halt disease evolution.

### 3.5 Stepwise dominance without irreversibility

While the pathogenic loop is continuous, dominant biological drivers may shift over time. This stepwise dominance is summarized in **Table 2**, which integrates vascular, immune and fibrotic mechanisms across disease phases.

**Table 2:** Stepwise mechanistic integration of vascular, immune and fibrotic pathways.

Phase dominant mechanism key mediators	Outcome
Vasospasm- Hypoxia–reperfusion stress ROS, NO↓, ET-1↑	Endothelial dysfunction
Altered lipid handling with oxLDL- accumulation; MCP-1, M-CSF	Inflammation initiation
Leukocyte adhesion and cytokine release (TNF-α, IL-6, IL-1, IFN-γ)	Immune amplification
EndoMT transition and fibroblast activation; TGF-β2, IL-4, IL-17, PDGF	Collagen synthesis
Fibrosis and structural remodeling; VEGF, Endothelin, TGF-β	Advanced structural sclerosis

Detailed stepwise representation of the dynamic pathogenic loop underlying systemic sclerosis (SSc), illustrating sequential yet overlapping phases of disease evolution. The phase-specific mechanisms summarized here are derived from convergent clinical, imaging and immunological studies.

Step 1: Early endothelial dysfunction and microvascular rarefaction driven by vasospasm, hypoxia–reperfusion stress, oxidative injury and vasoactive mediators, leading to vascular instability.

Step 2: Initiation of immune activation with recruitment of innate and adaptive immune cells, accompanied by lipid dysregulation and pro-inflammatory signaling.

Step 3: Cytokine-driven amplification of inflammation and sustained endothelial–immune crosstalk, reinforcing vascular injury and immune persistence.

Step 4: Transition toward fibrosis through endothelial–mesenchymal transition, fibroblast activation and extracellular matrix production.

Step 5: Structural tissue remodeling and advanced sclerosis, which further reinforce vascular damage and immune activation, closing a non-linear, self-sustaining pathogenic loop.

Importantly, these phases:

- overlap rather than replace each other,
- do not imply strict temporal irreversibility,
- allow re-entry points into the loop at multiple levels.

The sequential yet overlapping dominance of vascular, immune and fibrotic mechanisms across disease evolution is summarized in **Table 2**.

This scheme highlights the concept of stepwise dominance without strict irreversibility, emphasizing that pathogenic phases overlap, interact and allow re-entry into the loop at multiple levels, with important implications for stage-adapted and combinatorial therapeutic strategies.

This dynamic interpretation reconciles early vascular phenotypes, inflammatory-dominant presentations and rapidly fibrotic courses as different equilibrium states within the same system rather than distinct diseases entities.

### 3.6 Conceptual validation across clinical phenotypes

When mapped onto clinical observations, the model demonstrates strong explanatory coherence:

- Vascular-dominant phenotypes correspond to early loop configurations driven by endothelial instability.
- Inflammatory phenotypes reflect cytokine network expansion and immune geometry stabilization.
- Fibrotic phenotypes represent matrix-driven reinforcement of the loop, with reduced reversibility.

This interpretation aligns with longitudinal clinical observations indicating that SSc phenotypes reflect dynamic, partially reversible disease trajectories rather than fixed categorical entities (23).

Thus, clinical heterogeneity emerges as a function of axis weighting and temporal dynamics, not separate pathogenic entities.

### 3.7 Summary of results

Collectively, these results demonstrate that:

1. SSc operates as a self-reinforcing pathogenic loop.
2. Vascular dysfunction, immune dysregulation and fibrosis are structurally inseparable.
3. Immune persistence is governed by cytokine network geometry rather than by single mediators.
4. Fibrosis functions as an active signaling axis, not a passive endpoint.
5. Clinical heterogeneity reflects dynamic equilibrium states within the same pathogenic system.

These findings provide the conceptual foundation for the Discussion section, where translational and therapeutic implications are explored.

## 4 Discussions

### 4.1 Reframing SSc as a dynamic loop rather than a linear cascade

The present manuscript does not aim to provide an exhaustive review of the most recent literature, but rather to formalize a clinically grounded conceptual model integrating vascular, immune and fibrotic processes observed over time in SSc.

One of the persistent limitations of systemic SSc research over the past two decades has been the fragmentation of pathogenetic narratives. Vascular injury, immune dysregulation and fibrosis have frequently been described either as parallel processes or as sequential events depending on disciplinary focus. While each approach has generated valuable insights, none fully explains the defining clinical features of SSc: disease persistence, phenotypic heterogeneity, partial therapeutic responses and the frequent dissociation between inflammatory activity and fibrotic progression.

Taken together, these observations suggest that systemic sclerosis persistence is not driven by isolated pathogenic pathways, but by the stability of a self-reinforcing network architecture that resists collapse despite targeted perturbation.

The integrative framework proposed in this work reframes SSc as a self-sustaining pathogenic loop rather than a unidirectional cascade. In this model vascular dysfunction, immune activation and fibrosis are not temporally isolated stages but continuously interacting domains, each capable of re-entering and amplifying the others. This reconceptualization aligns more closely with real-world clinical trajectories in which early vascular abnormalities coexist with immune activation and fibrotic progression may advance despite apparent inflammatory quiescence.

Importantly, the loop model does not negate previous hypotheses but organizes them into a coherent dynamic architecture offering a higher-order explanation for disease behavior across stages.

### 4.2 Vascular dysfunction as an active immunomodulatory platform

Endothelial injury has long been recognized as an initiating hallmark of SSc. However, its role has often been framed as *passive* or *downstream* of immune aggression. The present model assigns a more central and active role to the vasculature, positioning endothelial dysfunction as a persistent immunomodulatory platform rather than a transient initiating event.

Endothelial cells under chronic stress exhibit altered nitric oxide bioavailability, increased endothelin-1 production and upregulation of adhesion molecules. These changes actively shape immune cell trafficking, antigen presentation thresholds and cytokine gradients. Consequently, vascular dysfunction is not merely permissive for immune activation but actively sculpts immune topology, contributing to the stability of pathogenic immune configurations.

This perspective helps explain why microvascular pathology persists even when systemic inflammatory markers fluctuate and why vascular-targeted therapies may exert immunomodulatory effects beyond hemodynamic control.

### 4.3 Immune geometry: beyond cytokine dominance

A central conceptual contribution of this work is the introduction of immune geometry as an organizing principle for immune dysregulation in SSc. Traditional models often emphasize dominant cytokines or helper T-cell subsets. However, such linear hierarchies struggle to account for redundancy, compensation and context-dependent signaling observed in clinical practice (13).

Within this framework, immune activity is better understood as a dynamic redistribution of signaling weight across interconnected axes rather than as the dominance of individual cytokines.

Within the geometric framework illustrated in **Figure 2**, cytokines and immune subsets are understood as interacting axes within a multidimensional network where pathogenic dominance emerges from spatial and functional relationships rather than absolute cytokine levels (9, 16, 26). Th17-associated pathways, Th2 profibrotic signaling, impaired regulatory buffering and interferon-mediated vascular effects coexist in dynamically stable configurations.

This organization explains why targeted inhibition of a single mediator frequently results in partial or transient benefit: the network adapts internally redistributing pathogenic weight without collapsing the loop.

This geometric representation is intended as a conceptual abstraction rather than a strict mechanistic map.

### 4.4 Th17 signaling as a structural stabilizer within the pathogenic loop

Within the proposed loop architecture Th17-associated signaling occupies a distinctive position, not as a primary driver of disease initiation but as a structural stabilizer that reinforces loop persistence once established. Clinical and experimental data suggest that IL-17–related pathways intersect vascular injury, innate immune activation and fibroblast responsiveness, thereby contributing to loop coherence rather than isolated inflammatory output (9, 13).

Importantly, Th17 signaling in this framework is not interpreted in isolation or as uniformly dominant across all disease stages. Instead, its relevance emerges from its capacity to bridge immune activation with endothelial dysfunction and matrix remodeling, particularly in intermediate disease phases. This positioning is consistent with variable therapeutic responses observed with IL-17 pathway modulation and supports the notion that efficacy may depend on timing and loop configuration rather than cytokine abundance alone.

By embedding Th17 activity within immune geometry rather than elevating it as a singular pathogenic axis the model avoids cytokine-centric reductionism while preserving biologically and clinically meaningful roles for Th17-mediated interactions.

### 4.5 Fibrosis as an active signaling compartment within the loop

Fibrosis in SSc is traditionally conceptualized as the terminal consequence of immune activation and vascular injury. Within the loop-based framework, however, fibrotic tissue is repositioned as an active signaling compartment that participates in sustaining disease dynamics rather than representing an irreversible endpoint.

Activated fibroblasts and myofibroblasts interact bidirectionally with immune and vascular components through cytokine production, matrix-derived signaling and mechanical feedback. Extracellular matrix remodeling alters tissue stiffness, cellular migration and endothelial behavior, thereby reinforcing immune activation and perpetuating vascular dysfunction. In this context, fibrosis functions as a stabilizing element of the pathogenic loop rather than a passive scar.

This reinterpretation helps explain why fibrotic progression may continue despite apparent control of inflammatory activity and why antifibrotic interventions alone often fail to modify broader disease trajectories unless integrated within a stage-adapted strategy.

### 4.6 Clinical heterogeneity as differential axis weighting within the pathogenic loop

Clinical heterogeneity is a defining feature of SSc (SSc) encompassing variability in organ involvement, disease tempo and therapeutic responsiveness (5, 9). Within the proposed loop-based framework this heterogeneity is not interpreted as evidence for distinct pathogenic entities but rather as the consequence of differential weighting of vascular, immune and fibrotic axes within a shared dynamic architecture.

Patients may present with loop configurations dominated by early and persistent vascular dysfunction, others with immune-driven amplification or with an early stabilization of fibrotic signaling. These configurations can coexist or shift over time reflecting changes in axis dominance rather than linear disease progression. Importantly, this interpretation accommodates both limited and diffuse phenotypes without requiring rigid categorical separation.

By conceptualizing heterogeneity as axis-weighting rather than disease fragmentation the model provides a coherent explanation for divergent clinical trajectories observed among patients with similar serological profiles or classification criteria. It also clarifies why therapeutic responses vary widely, as interventions targeting a single axis may have differential impact depending on the prevailing loop configuration at the time of treatment.

### 4.7 Operationalization and testable implications of the loop-based model

To move beyond a purely conceptual construct, the proposed loop framework requires operational anchors that can be assessed longitudinally and integrated into clinical observation. This axis-based interpretation aligns with emerging systems-immunology and network-oriented therapeutic frameworks, emphasizing combination, timing and context-dependent intervention rather than isolated target suppression (16, 19, 20).

Rather than relying on single biomarkers, operationalization is achieved by mapping dominant axis activity across vascular, immune and fibrotic domains, as summarized in **Table 2**. Each domain is defined by measurable clinical or paraclinical features that reflect relative axis weighting at a given disease stage.

Within this structure, testable implications emerge from shifts in axis dominance over time. Early vascular predominance may be reflected by persistent microvascular abnormalities and endothelial dysfunction preceding overt immune amplification whereas immune-dominant configurations may coincide with cytokine imbalance and enhanced immune–vascular crosstalk. Fibrotic stabilization, in turn, reflects matrix-driven signaling that persists despite partial attenuation of inflammatory markers. Importantly, these configurations are not necessarily sequential and may overlap, reinforcing the pathogenic loop rather than resolving it.

From a therapeutic perspective this model predicts that interventions targeting a single axis will have variable impact depending on the prevailing loop configuration at the time of treatment. Clinical improvement without sustained disease modification, partial responses or delayed progression can thus be interpreted as expected outcomes of axis-specific modulation within a resilient, self-reinforcing system. Consequently, the framework supports stage-adapted and combinatorial strategies aimed at attenuating reinforcing interactions between axes rather than suppressing isolated mediators (30). While it does not prescribe specific treatment algorithms, it provides a conceptual basis for aligning therapeutic timing and targeting with disease dynamics.

This perspective reframes therapeutic response not as pathway inhibition, but as the capacity to shift the system out of a stable pathogenic equilibrium.

### 4.8 Comparative perspective: distinct loop geometries in SSc, lupus and dermatomyositis

Placing SSc within a broader autoimmune context highlight both shared mechanisms and disease-specific configurations of immune-mediated injury (31). While vascular dysfunction, immune activation and tissue damage are common themes across systemic autoimmune diseases their geometric organization within pathogenic loops differs substantially.

In systemic lupus erythematosus immune dysregulation and autoantibody-driven inflammation dominate the loop with downstream multiorgan involvement affecting renal, neurological, hematological and cutaneous systems (30). When present, fibrotic processes are typically secondary to chronic immune injury rather than stabilizing elements of the loop itself. By contrast, dermatomyositis is characterized by a loop centered on immune-mediated microvascular and muscle injury where tissue damage predominates but does not generally evolve into progressive fibrosis (32).

SSc is distinguished by the early and persistent integration of vascular injury with immune activation and fibrotic stabilization resulting in a self-reinforcing loop in which fibrosis functions as an active signaling compartment rather than a terminal outcome. This comparison underscores that disease identity may be defined not by isolated pathways but by the relative weighting and coupling of pathogenic axes.

Such a perspective supports the notion that therapeutic strategies may be aligned with disease-specific loop geometry rather than extrapolated across autoimmune conditions based solely on shared molecular targets.

Taken together, this work frames SSc as a self-sustaining pathogenic loop governed by immune geometry, axis dominance and feedback reinforcement. While this conceptual model integrates diverse clinical and mechanistic observations its value ultimately depends on empirical validation and translational applicability. These considerations naturally lead to the future directions outlined below.

Accordingly, the value of the proposed framework lies in its capacity to organize heterogeneous clinical and biological observations into testable trajectories rather than in cataloguing recent molecular discoveries.

## 5 Future directions and research perspectives

The conceptual framework proposed here is intended as an integrative, hypothesis-generating scaffold rather than a finalized mechanistic model. Its primary value lies in organizing heterogeneous clinical and biological observations into a coherent loop-based architecture that can be empirically tested and refined.

To clearly delineate the scope of the model and to guide future validation its current limitations and prospective research directions are outlined below.

### 5.1 Limitations of the model

The framework is not intended to replace existing classification systems or clinical algorithms.

The dynamic vascular–immune–fibrotic loop is derived from longitudinal clinical observation, mechanistic reasoning, capillaroscopic interpretation and synthesis of existing experimental and clinical literature. It does not provide direct quantitative validation through prospective cohorts, multi-omics datasets or interventional trials (10). The model should therefore be interpreted as a conceptual organizing framework rather than a definitive causal proof.

The proposed immunological geometry is intentionally inferential and symbolic, designed to capture functional dominance, redundancy and spatial–temporal coupling among immune axes. While this approach reflects biological complexity more faithfully than linear hierarchies, it necessarily simplifies multidimensional immune processes influenced by genetic background, epigenetic regulation, metabolic state and environmental factors.

Although capillaroscopy is incorporated as a dynamic mirror of loop activity, the model does not yet integrate quantitative microvascular metrics with molecular immune signatures within a unified dataset. Such integration represents a critical step for future validation.

Finally, the present framework focuses primarily on SSc and may not be directly generalizable to other autoimmune or fibrotic diseases without disease-specific adaptation. Nonetheless, the loop-based logic and geometric interpretation may serve as a transferable conceptual template.

### 5.2 Future directions and research perspectives

This model opens several concrete avenues for future investigation.

#### 5.2.1 Longitudinal loop profiling

Prospective studies integrating nailfold videocapillaroscopy, circulating cytokine panels, immune cell phenotyping and fibrosis biomarkers could enable real-time mapping of loop dominance and transitions across disease stages.

#### 5.2.2 Immune geometry validation

Systems-immunology approaches—including single-cell RNA sequencing, spatial transcriptomics and computational network modeling—could be used to test whether immune activity in SSc organizes into stable geometric configurations rather than fluctuating linear cascades.

#### 5.2.3 Therapeutic geometry modulation

Rather than targeting isolated cytokines, future clinical trials could explore sequential or combinatorial strategies aimed at:

- disrupting vascular–immune coupling,
- restoring regulatory balance,
- attenuating fibrotic feedback simultaneously or in a stage-adaptive manner.

#### 5.2.4 Cross-disease extension

The conceptual architecture proposed here may be adapted to other immune-fibrotic diseases (e.g., lupus, interstitial lung disease, inflammatory myopathies), allowing comparative analysis of disease-specific loop geometries and shared pathogenic principles.

Taken together, these future directions emphasize that the primary contribution of the present work lies not in proposing a finalized mechanistic pathway but in offering a loop-based conceptual framework capable of integrating clinical heterogeneity, immune network behavior and therapeutic response. This perspective provides the foundation for the concluding synthesis and highlights the broader implications of immune geometry for understanding and modulating systemic autoimmune disease.

## 6 Conclusions

This work proposes a loop-based conceptual framework in which SSc emerges as a dynamic, self-sustaining system defined by continuous interaction between vascular dysfunction, immune dysregulation and fibrotic remodeling. Rather than reflecting a linear cascade, disease behavior arises from the stability and reconfiguration of a pathogenic network in which each domain reinforces and reshapes the others over time.

By integrating immune geometry with clinically observable domains—including nailfold videocapillaroscopy, inflammatory profiling and fibrotic trajectories—the model provides a structured platform for interpreting disease heterogeneity and therapeutic variability. Within this architecture, clinical outcomes are not determined solely by pathway activity, but by the relative dominance and coupling of system-level interactions.

Importantly, this framework positions fibrosis as an active signaling component and immune activity as a spatially organized network rather than a collection of isolated mediators. This shift from pathway-based to architecture-based thinking offers a unifying perspective that may better explain disease persistence and the limited durability of single-target interventions.

As a conceptual scaffold, the model is intended to support longitudinal evaluation, hypothesis generation and alignment of therapeutic strategies with dynamic disease states. Beyond SSc, this approach may provide a transferable framework for understanding immune-mediated diseases characterized by persistent, self-reinforcing network behavior. The broader conceptual implications of this framework have been further developed in a dedicated monographic work (33).

Ultimately, systemic sclerosis may be less a disease of individual pathways and more a disease of network stability—one that demands not stronger inhibition, but smarter reconfiguration.
